# The Sensitivity of Diagnostic Criteria of Marais S, et al. in Confirmed Childhood Tuberculous Meningitis

**DOI:** 10.3389/fped.2022.832694

**Published:** 2022-02-16

**Authors:** Mao-Shui Wang, Jun-Li Wang, Xin-Jie Liu, Yan-An Zhang

**Affiliations:** ^1^Department of Pediatrics, Qilu Hospital, Cheeloo College of Medicine, Shandong University, Jinan, China; ^2^Department of Lab Medicine, Shandong Provincial Chest Hospital, Cheeloo College of Medicine, Shandong University, Jinan, China; ^3^Department of Lab Medicine, Shandong Public Health Clinical Center, Cheeloo College of Medicine, Shandong University, Jinan, China; ^4^Shandong Key Laboratory of Infectious Respiratory Disease, Jinan, China; ^5^Department of Lab Medicine, The Affiliated Hospital of Youjiang Medical University for Nationalities, Baise, China; ^6^Department of Cardiovascular Surgery, Shandong Public Health Clinical Center, Cheeloo College of Medicine, Shandong University, Jinan, China

**Keywords:** children, tuberculous meningitis, diagnosis, criteria, diagnostic score

## Abstract

**Background:**

To establish the sensitivity of the diagnostic criteria published by Marais and co-workers in 2010 for childhood tuberculous meningitis (TBM), a retrospective study on children with confirmed TBM was conducted.

**Methods:**

Between January 2006 and December 2019, children consecutively diagnosed with TBM were recruited retrospectively at our center. TBM was defined in cases where any of the following criteria were met: the presence of acid-fast bacilli (AFB) in cerebrospinal fluid (CSF) microscopy, CSF nucleic acid amplification test (NAAT, +), or *M.tuberculosis* cultured from CSF. The demographic and clinical features of all enrolled patients were recorded including clinical characteristics, CSF findings, cerebral imaging features, and other evidence of TB.

**Results:**

A total of 30 children with confirmed diagnosis of TBM over an 14-year period were recruited. The mean age of patients was 7.2 ± 5.1 years and 16 (53.3%) were male. The estimated mean diagnostic score was 12.7 ± 2.4. Twenty-three (76.7%; 95% CI: 59.1–88.2%) patients were classified as “probable TBM” according to the Marais criteria and 7 (23.3%; 95% CI: 11.8–40.9%) as “possible TBM.” Further statistical analysis revealed significant differences in CSF scores between probable and possible TBM groups. Other variables reported at a relatively low frequency, such as symptoms and imaging features, made little contribution to TBM diagnosis according to the Marais criteria.

**Conclusion:**

Childhood TBM could be effectively identified by the criteria defined by Marais et al. However, further revision is required to ensure that the system is more sensitive and easier to perform in practice.

## Introduction

Tuberculous meningitis (TBM) is an ongoing serious threat to child health. In China, 9.4% of children with tuberculosis (TB) are diagnosed with TBM, with 14.7% showing poor outcomes, such as death and transferred to a high care center (1, 2). Diagnostic delay is an important contributor to mortality in childhood-associated TBM (3). It was reported that about one-fifth of TBM children were asymptomatic on admission, and the sensitivities of Ziehl–Neelsen staining, culture, and polymerase chain reaction (PCR) for detection of childhood TBM were 25, 58, and 66.3%, respectively (4). Furthermore, in childhood TB, interferon-gamma release assay (IGRA) has a good sensitivity (83.3%) and few indeterminate results (2.5%) were reported (5). However, in childhood TBM, a relative low sensitivity of 71.7% was reported and indeterminate results (17.0%) were common (6). Moreover, limited techniques for effective TBM diagnosis in children, such as Xpert Ultra, are currently available (7). Two key factors are responsible for the diagnostic limitations in childhood TBM: (1) cerebrospinal fluid (CSF) is a paucibacillary specimen type and (2) TB in children is often paucibacillary. In addition, TBM is difficult to treat and often responds poorly to conventional TB treatment (8, 9).

TBM infection is easy to confirm, usually based on microbiological evidence in CSF samples. Routine microbiological methods include acid-fast bacilli (AFB) smear, culture, and nucleic acid amplification test (NAAT). However, during the past few decades, the definitions of “possible” and “probable” TBM have varied widely among studies. These inconsistencies induce selection bias and have a significant impact on the accuracy of systematic reviews (10). In 2010, a standardized set of diagnostic criteria for TBM was introduced by Marais et al. (11). Since then, this diagnostic scoring system has been widely used in the corresponding field and cited in numerous clinical studies. The Marais classification criteria are based on Thwaites' Index, which has previously shown good performance (12–14). However, these criteria are associated with a number of disadvantages that need to be addressed: (1) the difference between childhood and adult TBM is not fully considered, (2) the scoring system is presented as a personal view and lacks sufficient data to support its efficiency, and (3) insufficient information is available on the utility of the criteria in China.

This retrospective study aimed to evaluate the efficiency of the diagnostic scoring system in confirmed childhood TBM. Our findings should aid in improving application of the criteria for childhood TBM and providing further evidence to tailor the scoring system for more accurate diagnosis of TBM in children.

## Methods

This study was conducted at the Shandong Provincial Chest Hospital in keeping with the Declaration of Helsinki. The study protocol was approved by the Ethical Committee of Shandong Provincial Chest Hospital (approval No. 2020XKYYEC-29). Due to the retrospective nature of this study and the anonymous nature of data analyzed, written informed consent was waived by the Ethical Committee of Shandong Provincial Chest Hospital.

Between January 2006 and December 2019, consecutive patients ( ≤ 15 years old) with confirmed diagnosis of TBM were retrospectively recruited for study. TBM was defined in cases where any of the following criteria were met: acid-fast bacilli (AFB) in cerebrospinal fluid (CSF) microscopy, CSF NAAT (+) or *M.tuberculosis* cultured from CSF. The demographic characteristics and clinical features of all enrolled patients were recorded using standardized questionnaires developed on the basis of four criteria, including clinical, CSF and cerebral imaging features, and other evidence of TB (Table 1). Subsequently, a diagnostic score was calculated and patients were divided as probable and possible TBM based on the Marais criteria (11). Probable TBM is defined if a diagnostic score is ≥ 12 (with imaging) or ≥ 10 (without imaging). Possible TBM is defined if a diagnostic score is 6–11 (with imaging) or 6–9 (without imaging).

**Table 1:** Diagnostic items and corresponding scores for tuberculous meningitis classification.

**Variables**		**Diagnostic score**
**Clinical criteria (Maximum** **=** **6)**		
	Symptom duration (>5 days)	4
	Symptoms (at least 1 symptom): weight loss (or poor weight gain in children), night sweats, and cough (>2 weeks)	2
	TB experience: TB contact, positive TST or IGRA	2
	Focal neurological deficit (excluding cranial nerve palsies)	1
	Cranial nerve palsy	1
	Altered consciousness	1
**CSF criteria (Maximum** **=** **4)**		
	Clear appearance	1
	Cells (10–500/μl)	1
	Lymphocytic predominance (>50%)	1
	Protein (>1 g/L)	1
	CSF/plasma glucose (<50%) or CSF glucose (<2.2 mmol/L)	1
**Cerebral imaging criteria (Maximum** **=** **6)**		
	Hydrocephalus	1
	Basal meningeal enhancement	2
	Tuberculoma	2
	Infarct	1
	Pre-contrast basal hyperdensity	2
**Evidence of tuberculosis elsewhere (Maximum** **=** **4)**		
	Chest radiograph	2 or 4
	TB signs	2
	or Miliary TB	4
	Radiaographical evidence for TB outside the CNS	2
	Microbiological evidence (extra-neural specimen)	
	AFB or mycobacterial culture	4
	NAAT	4

*TB, tuberculosis; TST, tuberculin skin test; IGRA, interferon gamma release assay; CSF, cerebrospinal fluid; CNS, central nervous system; NAAT, nucleic acid amplification testing*.

Continuous data were presented as mean ± standard deviation (SD) and categorical data as counts (percentages). In addition, 95% confidence interval (CI) was calculated whenever appropriate. Student's *t-*test was used to compare mean values of diagnostic scores (including total diagnostic score and scores for clinical criteria, CSF criteria, cerebral imaging criteria, and evidence of tuberculosis elsewhere) between two groups and *P* < 0.05 considered statistically significant.

## Results

A total of 149 childhood TBM cases diagnosed over the past 14 years were included for analysis, 30 of which were confirmed based on CSF culture (+, *n* = 24), CSF AFB (+, *n* = 2), and NAAT (+, *n* = 16). Among the 30 children, one patient has a positive result of CSF AFB smear, 5 have positive results of CSF PCR, 12 have positive results of CSF culture, one has positive results of CSF smear and culture, and 11 have positive results of CSF culture and PCR. The mean age was 7.2 ± 5.1 years and 16 (53.3%) were male. Clinicopathological characteristics were divided into four sets, shown in Table 2.

**Table 2:** The clinicopathological of confirmed pediatirc tuberculous meningitis in Shandong, China.

**Variables**			***N* (%)**	**Mean (SD)**
Number			30	
Sex, (%, Male)			16 (53.3%)	
Age (years)			7.2 ± 5.1	
Clinical criteria				
	Symptom duration (>5 days)		30 (100%)	27.0 ± 34.2 days
	Symptoms			
		Weight loss	1 (3.3%)	
		Night sweats	0 (0%)	
		Cough (>2 weeks)	6 (20.0%)	
	TB experience			
		Close TB contact	10 (33.3%)	
		Positive TST or IGRA	12 (12/15, 80.0%)	
	Focal neurological deficit			
		Cranial nerve palsy	0 (0%)	
		Cranial nerve palsy (excluding)	5 (16.7%)	
	Altered consciousness		4 (13.3%)	
CSF criteria				
	Clear appearance		25 (83.3%)	
	Cells (10–500/μl)		27 (90.0%)	201.1 ± 174.2 /μl
	Lymphocytic predominance (>50%)		19 (19/28, 67.9%)	64.5 ± 25.5 %
	Protein (>1 g/L)		13 (43.3%)	1,240 ± 1,048 g/L
	CSF/plasma glucose(<50%) or CSF glucose (<2.2 mmol/L)		26 (86.7%)	1.64 ± 0.88 mmol/L
**Cerebral imaging criteria**			
	Hydrocephalus		7 (7/24, 29.2%)	
	Basal meningeal enhancement		1 (1/24, 4.2%)	
	Tuberculoma		13 (13/24, 54.2%)	
	Infarct		0 (0/24, 0%)	
	Pre-contrast basal hyperdensity		5 (5/24, 20.8%)	
**Evidence of tuberculosis elsewhere**			
	Chest radiograph			
		TB signs	10 (10/27, 37.0%)	
		Miliary TB	13 (13/27, 48.2%)	
	Other radiaographical evidence for TB (excluding CNS and chest)		0 (0/3, 0%)	
	Microbiological evidence (extra-neural specimen)			
		AFB	2 (2/10, 20.0%)	
		Mycobacertial culture	2 (2/10, 20.0%)	
		NAAT	2 (2/9, 22.2%)	

*TB, tuberculosis; TST, tuberculin skin test; IGRA, interferon gamma release assay; CSF, cerebrospinal fluid; CNS, central nervous system; NAAT, nucleic acid amplification testing*.

The mean symptom duration was 27.0 ± 34.2 days, with all symptoms recorded for over 5 days. Ten patients had a history of close TB contact and 12 were confirmed with TB infection via tuberculin skin test (TST) or interferon gamma release assay (IGRA). Common TB symptoms, such as weight loss (*n* = 1, 3.3%), night sweats (0, 0%), and cough (>2 weeks; *n* = 6, 20%), were reported in a minority of patients. In addition, focal neurological deficits (excluding cranial nerve palsy) and altered consciousness were recorded in 5 and 4 patients, respectively.

Clear appearance of CSF was reported in 25 cases (25/30, 83.3%). Among these, 19 patients (19/28, 67.9%) showed lymphocyte-predominant CSF accumulation (lymphocyte proportion: 64.5 ± 25.5%), 27 (90%) had cell numbers between 20 and 500/μL (mean level of 201.1 ± 174.2/μL), and 26 (86.7%) presented with a low CSF/plasma glucose ratio (<50%) or CSF glucose (<2.2 mmol/L; mean, 1.64 ± 0.88 mmol/L). However, CSF protein content above 1 g/L (concentration, 1,240 ± 1,048 mg/L) was observed in only half of the patients.

Twenty-four patients underwent radiological examinations. Hydrocephalus, basal meningeal enhancement, tuberculoma, infarct, and pre-contrast basal hyperdensity were reported in 7 (29.2%), 1 (4.2%), 13 (54.2%), 0 (0%), and 5 (20.8%) patients, respectively.

Twenty-seven patients were subjected to chest radiography, which revealed TB signs and miliary TB in 10 (37.0%) and 13 (48.2%) patients, respectively. Extra-neural specimens were subjected to microbiological tests. Overall, two patients were smear-positive, two were culture-positive, and two were NAAT-positive.

The diagnostic score was calculated for all patients as follows: 10 (*n* = 6), 11 (*n* = 4), 12 (*n* = 8), 13 (*n* = 2), 14 (*n* = 3), 15 (*n* = 1), 16 (*n* = 5), and 19 (*n* = 1). The mean score was subsequently estimated as 12.7 ± 2.4. Twenty-three patients (76.7%; 95% CI: 59.1–88.2%) were classified as probable TBM according to the Marais criteria (with imaging, >12; without imaging, >10) and 7 (23.3%; 95% CI: 11.8–40.9%) as possible TBM. Further statistical analysis showed significant differences in CSF scores between probable and possible TBM groups (Table 3). The collective data clearly indicate that more attention should be paid to improvement of diagnostic criteria based on CSF findings.

**Table 3:** Diagnostic scores of four sets of TBM diagnositc criteria between probable and possible TBM.

**Variables**	**Possible TBM**	**Probable TBM**	***P*-values**
Number	7	23	
Clinical criteria	5.14 ± 0.90	5.48 ± 0.85	0.403
CSF criteria	2.86 ± 1.07	3.61 ± 0.72	0.04
Cerebral imaging criteria	0.85 ± 1.21	1.70 ± 1.74	0.247
Evidence of tuberculosis elsewhere	1.71 ± 1.80	2.61 ± 1.53	0.204
Diagnostic scores	10.57 ± 0.53	13.40 ± 2.33	<0.001

*TBM, tuberculous meningitis; CSF, cerebrospinal fluid*.

## Discussion

TBM is the most severe form of TB and a significant cause of morbidity and mortality worldwide. Clinical diagnosis of TBM remains a significant challenge in practice, since a definitive diagnosis is hard to establish and a uniform clinical case definition is absent. In 2010, a case definition of TBM was published by Marais et al. (11). Over the last decade, these criteria have been cited more than 700 times and performed well in clinical research. In the current study, we assessed the utility of the Marais criteria in classification of childhood TBM. Although further revisions are required, our data support the high efficiency and utility of the Marais criteria for definitive diagnosis of childhood TBM.

Fever should be introduced into the current guidelines and the scores of items of clinical criteria refined. In our study, fever was the most common symptom, reported in 29 patients (96.7%, data not shown). Previously, fever was identified as a protective factor for the treatment delay of TBM (15). Therefore, introduction of fever into the current criteria may improve diagnostic performance and improve management of childhood TBM. In addition, based on the observation that a high proportion of TBM subjects have prior TB diagnosis and long duration of illness but relatively low frequency of other clinical presentations, we conclude that items of clinical criteria should be optimized and score values further refined, especially for meningitis symptoms.

CSF features are widely utilized for classification as possible TBM but associated with a number of limitations. First, the variable CSF scores between probable and possible TBM cannot be explained by a single CSF biomarker, since no differences are evident between the groups for each CSF feature. Second, atypical CSF findings reflect an abnormal state and could be used to evaluate severity of TBM. Similarly, the diagnostic score may correlate with progression of childhood TBM. Third, lymphocytic predominance and CSF protein have a relatively poor performance in classification. In our experiments, patients with lymphocytic predominance accounted about 70% of the study group. Although this percentage appears lower than that of other studies (~80%) (11), variable distribution is reported (16–18). Additionally, the criterion for CSF protein level <1 g/L is easily met in clinical practice and other references (such as 0.45 g/L and 0.8 g/L) are additionally used in clinical research (19–21). Fourth, chloride should be considered in the revised version of diagnostic criteria, since high CSF chloride levels are associated with treatment delay of TBM (15). The utility of CSF chloride in diagnosis of TBM has been previously evaluated (22).

Hydrocephalus, basal meningeal enhancement, infarct, and tuberculoma are the four main characteristics of TBM and the presence of these features on neuroimaging enhances confidence in clinical diagnosis (23–25). Neuroimaging is considered invaluable for assessment of TBM (26). In our study, these four features showed no sensitivity in TBM diagnosis. In addition, although tuberculoma was the most common presentation (54.2%), diagnosis was mostly made based on positive responses to anti-TB therapy. Another major limitation of this study was that several cases were confirmed based on microbiological evidence and radiological assessment was thus waived at the initial evaluation stage, leading to lack of imaging data. Overall, 66.7% of the subjects had pulmonary involvement and half the patients had miliary TB. These findings support the significance of respiratory specimens and need for caution in cases of miliary TB for potential diagnosis of childhood TBM.

Our study has several limitations that should be taken into consideration. First, comparison between childhood TBM and controls was not performed, since the hospital was a referral TB setting and few meningitis cases of other etiologies were admitted. Moreover, few TBM cases were confirmed over the long-term period of analysis. Second, due to the retrospective nature of the study, some data were overlooked during collection, which would decrease the actual diagnostic score and result in misclassification. Third, the lack of TST and IGRAs is another limitation, since their use might change the performance of the Marais criteria. Therefore, our results should be interpreted with caution. Fourth, due to other study limitations, such as single center design, small sample size, and lack of controls, further research is required to revise the criteria and evaluate the efficiency of both primary and revised versions in diagnosis of suspected childhood TBM.

In conclusion, although childhood TBM could be effectively identified by the Marais criteria, further revision of these guidelines is essential to improve diagnostic performance, reliability, and ease of use.

## Data Availability Statement

The original contributions presented in the study are included in the article/supplementary material, further inquiries can be directed to the corresponding author/s.

## Ethics Statement

The study protocol was approved by the Ethical Committee of Shandong Provincial Chest Hospital. Due to the retrospective nature of this study and the anonymous nature of data analyzed, written informed consent was waived by the Ethical Committee of Shandong Provincial Chest Hospital.

## Author Contributions

X-JL and Y-AZ designed the study and supervised data collection. M-SW collected data, performed statistical analysis, and drafted the initial manuscript. J-LW revised the manuscript. All authors approved the final version of the report.

## Conflict of Interest

The authors declare that the research was conducted in the absence of any commercial or financial relationships that could be construed as a potential conflict of interest.

## Publisher's Note

All claims expressed in this article are solely those of the authors and do not necessarily represent those of their affiliated organizations, or those of the publisher, the editors and the reviewers. Any product that may be evaluated in this article, or claim that may be made by its manufacturer, is not guaranteed or endorsed by the publisher.
